# Using Telehealth to Deliver Primary Care to Adolescents During and After the COVID-19 Pandemic: National Survey Study of US Primary Care Professionals

**DOI:** 10.2196/31240

**Published:** 2021-09-10

**Authors:** Melissa B Gilkey, Wei Yi Kong, Qian Huang, Brigid K Grabert, Peyton Thompson, Noel T Brewer

**Affiliations:** 1 Department of Health Behavior University of North Carolina at Chapel Hill Chapel Hill, NC United States; 2 Department of Pediatrics University of North Carolina at Chapel Hill Chapel Hill, NC United States

**Keywords:** adolescent health, primary care, telemedicine, health communication, health services, telehealth, adolescent, young adult, teenager, COVID-19, survey, policy, access

## Abstract

**Background:**

The COVID-19 pandemic has led to unprecedented use of telehealth, including by primary care professionals (PCPs) who serve adolescents.

**Objective:**

To inform future practice and policies, we sought to characterize PCPs’ recent experience using adolescent telehealth as well as their support for it after the COVID-19 pandemic is over.

**Methods:**

From February to March 2021, we conducted a web-based survey of 1047 PCPs in the United States. Our national sample included physicians (747/1047, 71%), advanced practice providers (177/1047, 17%), and nurses (123/1047, 12%) who provided primary care to adolescents aged 11-17 years.

**Results:**

Most PCPs reported using telehealth for a low, moderate, or high proportion of their adolescent patients in the three months prior to the survey (424/1047, 40%, 286/1047, 27%, and 219/1047, 21%, respectively); only 11% (118/1047) reported no use. A majority of respondents agreed that adolescent telehealth increases access to care (720/1047, 69%) and enables them to provide high-quality care (560/1047, 53%). Few believed that adolescent telehealth takes too much time (142/1047, 14%) or encourages health care overuse (157/1047, 15%). Most supported giving families the option of adolescent telehealth for primary care after the pandemic is over (683/1047, 65%) and believed that health insurance plans should continue to reimburse for telehealth visits (863/1047, 82%). Approximately two-thirds (702/1047, 67%) wanted to offer adolescent telehealth visits after the pandemic, with intentions being higher among those with recent telehealth experience (*P*<.001).

**Conclusions:**

PCPs in our national sample reported widespread use of and predominantly positive attitudes toward adolescent telehealth. Our findings also suggest broad support among PCPs for continuing to offer adolescent telehealth after the COVID-19 pandemic ends.

## Introduction

The COVID-19 pandemic has rapidly transformed health care delivery in the United States; for the first time, telehealth is playing a central role in the delivery of primary care for adolescents. Research conducted prior to the pandemic suggests that telehealth is a promising mode of delivering discrete types of health care for adolescents, including in the areas of mental health, asthma and diabetes management, and gender-affirming care [1-4]. A small body of research also documents the adolescent medicine community’s impressive efforts to rapidly scale up telehealth programs in the first months of the pandemic [5-7]. However, to our knowledge, no published national studies have explored the experience of primary care professionals (PCPs) in delivering care to adolescents in the ensuing period, during which telehealth has presumably become a standard offering for many. To address this gap, we surveyed a national sample of PCPs. Our aims were to characterize PCPs’ recent adolescent telehealth use and attitudes as well as their support for continuing to offer adolescent telehealth after the COVID-19 pandemic is over.

## Methods

### Participants and Procedures

We conducted a web-based survey of PCPs from February to March 2021. We contracted with a survey research company to administer the survey, which we developed, to a standing national panel. The company maintained the panel using a combination of recruitment methods, including web-based registration, referrals, marketing emails, and digital advertisements. As part of the recruitment process, physicians provided licensure information used to verify their identity. For our survey, eligible panel members were US physicians, advanced practitioners (ie, nurse practitioners and physician assistants), and nurses who provided primary care, including vaccinations, to adolescents aged 11-17 years. In compliance with state policies governing PCPs’ survey participation, our sample excluded residents of Vermont.

The survey company emailed invitations and up to two reminders to panel members. A total of 1055 panel members responded by accessing the survey. The response rate was 61% among physicians and 41% among advanced practitioners and nurses (American Association for Public Opinion Research response rate 4) [8]. Participants provided informed consent and received up to US $80 for their participation, depending on market rates in their area. Based on survey responses, we excluded 8 PCPs who indicated that they saw no adolescent patients in a typical week, resulting in a final sample size of 1047 participants. The University of North Carolina Institutional Review Board approved the study protocol.

### Measures

Our survey began with an introductory statement that defined adolescent telehealth as visits by videoconference or telephone for patients aged 11-17 years. The survey next assessed the extent of PCPs’ recent telehealth use with a closed-ended question on the proportion of adolescent patients they saw by telehealth in the 3 months prior to the survey; we recategorized responses as high (51%-75%, 76%-99%, and 100%), moderate (26%-50%), low (1%-25%), or no (0%) use. Among PCPs with any (>0%) use, the survey used 6 closed-ended items to assess telehealth practice. Of these items, 4 used pre-specified lists to assess the type of care provided, perceived advantages, perceived disadvantages, and technological barriers. One item assessed which vaccines PCPs always recommended during telehealth visits for adolescents who were due: seasonal influenza; human papillomavirus (HPV); tetanus, diphtheria, and acellular pertussis (Tdap); and meningococcal ACWY. One item assessed how often PCPs requested confidential time to speak with adolescents during telehealth visits.

Our survey assessed PCPs’ perceptions of adolescent telehealth with 7 closed-ended questions that used 5-point response scales, ranging from “strongly disagree” (1) to “strongly agree” (5). Of these items, 4 assessed PCPs’ attitudes on whether adolescent telehealth increases access to care, is a way they can provide high-quality care, takes too much time, or encourages health care overuse. A total of 2 items assessed PCPs’ support for adolescent telehealth after the COVID-19 pandemic is over in terms of whether families should still have the option to use telehealth for primary care visits and whether health insurance plans should continue to reimburse for visits. One item assessed PCPs’ intentions in terms of whether they wanted to offer adolescent telehealth visits once the pandemic is over. For all 7 items, we recategorized responses as disagree (“somewhat” or “strongly”), neither agree or disagree, or agree (“somewhat” or “strongly”).

Our survey assessed PCPs’ demographic and professional characteristics, including their training, gender, race, number of years in practice, and number of adolescent patients seen in a typical week (Table 1). The survey also assessed characteristics of the clinics in which the PCPs worked. These measures included clinic specialty (family medicine or pediatrics), practice type (solo/group vs other), whether the clinic was part of a health care system or network, the rurality of the area the clinic served, the US census region in which the clinic was located, and the extent to which the clinic experienced financial strain due to the COVID-19 pandemic.

**Table 1:** Sample characteristics (N=1047).

Characteristic			Value, n (%)
**Primary care professional characteristics**			
	**Training**		
		Physician	747 (71)
		Advanced practice provider^a^	177 (17)
		Nurse	123 (12)
	**Gender**		
		Woman	515 (49)
		Man	492 (47)
		Other^b^	40 (4)
	**Race**		
		White	717 (68)
		Black	41 (4)
		Asian	170 (16)
		Other	119 (11)
	**Years in practice**		
		0-9	252 (24)
		10-19	395 (38)
		20 or more	400 (38)
	**Adolescent patients seen in a typical week**		
		1-9	283 (27)
		10-24	431 (41)
		25 or more	333 (32)
	**Proportion of adolescents seen by telehealth in prior 3 months**		
		None (0%)	118 (11)
		Low (1%-24%)	424 (40)
		Moderate (25%-50%)	286 (27)
		High (51%-100%)	219 (21)
**Clinic or practice characteristics**			
	**Specialty**		
		Family medicine	748 (71)
		Pediatrics	299 (29)
	**Practice type**		
		Solo or group	696 (66)
		Other^c^	351 (34)
	**Part of a health care system**		
		No	457 (44)
		Yes	590 (56)
	**Rurality**		
		Urban	363 (35)
		Suburban	525 (50)
		Rural	159 (15)
	**Region**		
		Northeast	265 (25)
		Midwest	247 (24)
		South	333 (32)
		West	202 (19)
	**COVID-19–related financial strain**		
		None or a little	360 (34)
		Moderate to high	687 (66)

^a^Includes nurse practitioners and physician assistants.

^b^Includes neither woman nor man, prefer to self-describe, and prefer not to say.

^c^Includes hospital- and university-affiliated clinics, Federally Qualified Health Centers, and community, public health, and nonprofit clinics.

### Statistical Analysis

We used Pearson chi-square tests to compare the proportions of PCPs who indicated that they always recommended the seasonal influenza vaccine versus each of the other vaccines (HPV, Tdap, meningococcal) during adolescent telehealth visits. We compared the number of advantages and disadvantages that PCPs endorsed for adolescent telehealth using a Wilcoxon signed-rank test. We used bivariate logistic regression to identify correlates of PCPs’ intentions to offer adolescent telehealth visits once the COVID-19 pandemic is over, modeling the outcome as yes (“agree”) versus no (“neither agree or disagree” and “disagree”). We then simultaneously entered statistically significant correlates into a multivariable model. We conducted analyses using Stata, version 15.1 (StataCorp LLC). Statistical tests were two-tailed with a critical alpha of .05.

## Results

### Participant Characteristics

Our sample of 1047 respondents comprised physicians (n=747, 71%), advanced practitioners (n=177, 17%), and nurses (n=123, 12%; Table 1). Most had 10 or more years of experience in practice (795/1047, 76%) and saw 10 or more adolescent patients in a typical week (764/1047, 73%). PCPs worked in clinics focusing on family medicine (748/1047, 71%) or pediatrics (299/1047, 29%). The clinics were located in all four US regions; some were in clinical systems or networks (590/1047, 56%), and some served rural areas (159/1047, 15%). Two-thirds (687/1047, 66%) of PCPs reported that their clinics had experienced moderate to high financial strain due to the COVID-19 pandemic.

### Telehealth Practice

Almost all PCPs reported using telehealth to see adolescent patients in the 3 months prior to the survey (Table 1). Approximately one-fifth of our sample (219/1047, 21%) indicated high adolescent telehealth use, while others reported more moderate (286/1047, 27%) or low (424/1047, 40%) use. Only 11% of PCPs (118/1047) reported no recent adolescent telehealth use.

The 929 PCPs with recent telehealth visits most often used telehealth for chronic disease management (n=599, 64%), acute care (n=571, 61%), mental and behavioral health (n=561, 60%), or vaccine consultations (n=406, 44%) (Table 2). Most of these 929 PCPs indicated that they always recommended seasonal influenza vaccination during telehealth visits if adolescents were due (n=798, 86%), but somewhat fewer said the same for HPV, Tdap, and meningococcal vaccines (n=715, 77%, n=709, 76%, and n=612, 66%, respectively; all *P*<.001). Approximately one-quarter of these PCPs (263/929, 28%) reported that they “always” or “often” requested time to speak to adolescents confidentially during telehealth visits.

**Table 2:** Telehealth practice among primary care professionals with recent adolescent telehealth visits (n=929).

		Value, n (%)
**Type of adolescent telehealth visits provided**		
	Chronic disease management	599 (64)
	Acute care	571 (61)
	Mental and behavioral health	561 (60)
	Vaccine consultation	406 (44)
	Other well-child care	387 (42)
	Sexual health and contraceptive counseling	359 (39)
	None of these	14 (2)
**Vaccines always recommended if due**		
	Seasonal influenza	798 (86)
	Human papillomavirus	715 (77)
	Tetanus, diphtheria, and acellular pertussis	709 (76)
	Meningococcal	612 (66)
	None of these	48 (5)
**Request to speak with adolescent confidentially**		
	Always or often	263 (28)
	Sometimes	356 (38)
	Rarely or never	310 (33)
**Technology problems^a^**		
	Poor-quality internet connections	499 (85)
	Families’ lack of internet-enabled devices	353 (60)
	Lack of training for providers and staff	195 (33)
	Difficulty working with medical interpreters	128 (22)
	None of these	21 (4)

^a^Among the subset of participants who reported technology problems as a disadvantage (n=584).

**Figure 1:**
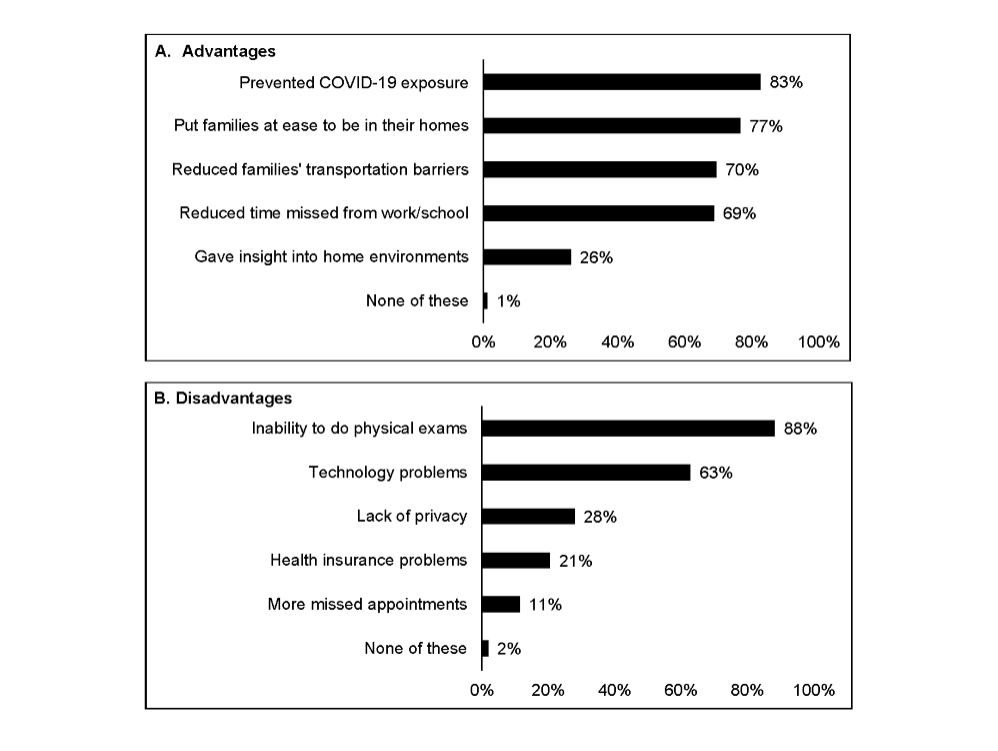
Perceived advantages (A) and disadvantages (B) of adolescent telehealth by primary care providers (n=929).

On average, PCPs reported more advantages than disadvantages of their recent adolescent telehealth use (mean=3.2 of 5 advantages, SD 1.3, vs 2.1 of 5 disadvantages, SD 0.9; *P*<.001). The most common advantages were preventing COVID-19 exposure (768/929, 83%), putting families at ease (712/929, 77%), and reducing families’ transportation (646/929, 70%) or time (639/929, 69%) burdens (Figure 1). Only approximately one-quarter of PCPs reported that gaining insight into families’ home environments was an advantage (243/929, 26%). The most common disadvantages were the inability to perform physical examinations (820/929, 88%) and technology problems (584/929, 63%). Only a minority of PCPs indicated that a lack of privacy (260/929, 28%), health insurance problems (191/929, 21%), or more missed appointments (106/929, 11%) were disadvantages. Of the 584 PCPs who indicated technology problems as a disadvantage, most reported that poor quality internet connections (n=499, 85%) and families’ lack of internet-enabled devices (n=353, 60%) were common barriers (Table 2).

### Telehealth Attitudes and Postpandemic Support

**Figure 2:**
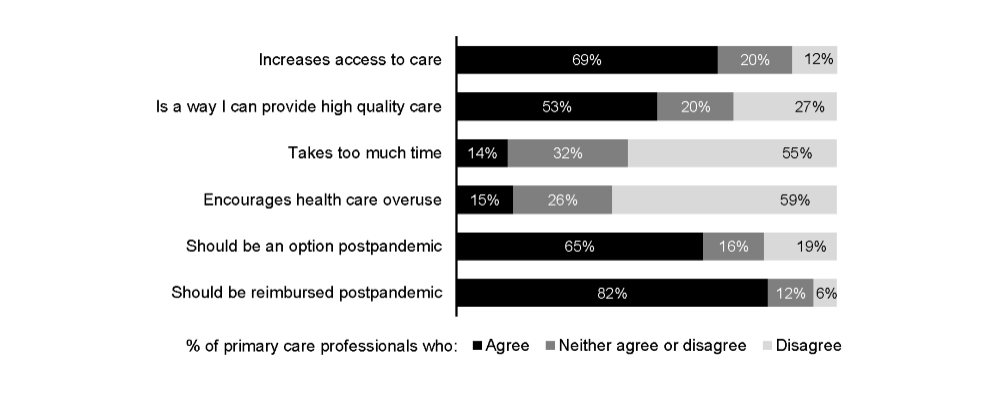
Primary care providers’ attitudes toward and support for postpandemic adolescent telehealth (n=1047).

Most PCPs reported positive attitudes toward adolescent telehealth. A majority agreed that telehealth increased access to care for adolescents (720/1047, 69%) and was a way they could provide high-quality care (560/1047, 53%, Figure 2). Few agreed that telehealth took too much time (142/1047, 14%) or encouraged families to overuse health care for adolescents (157/1047, 15%).

Most PCPs indicated support for adolescent telehealth after the COVID-19 pandemic is over (Figure 2). Approximately two-thirds (683/1047, 65%) agreed that families should continue to have the option of telehealth for adolescent primary care visits. Approximately four-fifths (863/1047, 82%) agreed that health insurance plans should continue to reimburse for telehealth visits.

### Telehealth Intentions

Approximately two-thirds of the 1047 PCPs agreed that they want to offer adolescent telehealth visits once the COVID-19 pandemic is over (n=702, 67%), while the remainder disagreed (n=188, 18%) or neither agreed nor disagreed (n=157, 15%). In multivariable analyses, wanting to offer telehealth was more common among PCPs with high, moderate, and low versus no experience seeing adolescents by telehealth in the 3 months prior to the survey (175/219, 80%, 211/286, 74%, and 280/424, 66%, vs 36/118, 31%, respectively; all *P*<.001) (Table 3). Wanting to offer telehealth was also more common among PCPs who worked in clinics that served urban versus rural areas (258/363, 71%, vs 94/159, 59%, *P*=.02) or that were located in the South or West regions versus the Northeast region (231/333, 69%, and 143/202, 71%, vs 161/265, 61%; *P*=.003 and .047, respectively). Wanting to offer telehealth was less common for PCPs with more years in practice (262/395, 66%, for 10-19 years and 246/400, 62%, for ≥20 years vs 194/252, 77%, for ≤9 years; both *P*=.02). In bivariate analyses, PCPs’ adolescent telehealth intentions correlated with working outside of a solo/group practice or within a health care system, but these associations did not retain statistical significance in the multivariable model.

**Table 3:** Correlates of PCPs’ intentions to offer adolescent telehealth after the COVID-19 pandemic (N=1047).

			PCPs^a^ who want to offer telehealth, n (%)	Bivariate analysis		Multivariable analysis	
				OR^b^ (95% CI)	*P* value	OR (95% CI)	*P* value
**PCP characteristics**							
	**Training**						
		Physician (n=747)	492 (66)	1	N/A^c^	—^d^	N/A
		Advanced practice provider (n=177)	126 (71)	1.28 (0.89-1.83)	.18	—	N/A
		Nurse (n=123)	84 (68)	1.12 (0.74-1.68)	.60	—	N/A
	**Years in practice**						
		0-9 (n=252)	194 (77)	1	N/A	1	N/A
		10-19 (n=395)	262 (66)	0.59 (0.41-0.84)	.004	0.63 (0.43-0.92)	.02
		20 or more (n=400)	246 (62)	0.48 (0.33-0.68)	<.001	0.64 (0.44-0.94)	.02
	**Adolescent patients seen in typical week**						
		1-9 (n=283)	190 (67)	1	N/A	—	N/A
		10-24 (n=431)	289 (67)	1.00 (0.72-1.37)	.98	—	N/A
		25 or more (n=333)	223 (67)	0.99 (0.71-1.39)	.96	—	N/A
	**Proportion of adolescents seen by telehealth in prior 3 months**						
		None (0%) (n=118)	36 (31)	1	N/A	1	N/A
		Low (1%-24%) (n=424)	280 (66)	4.43 (2.85-6.88)	<.001	4.61 (2.92-7.27)	<.001
		Moderate (25%-50%) (n=286)	211 (74)	6.41 (4.00-10.28)	<.001	6.53 (4.01-10.64)	<.001
		High (51%-100%) (n=219)	175 (80)	9.06 (5.43-15.13)	<.001	8.99 (5.31-15.23)	<.001
**Clinic or practice characteristics**							
	**Specialty**						
		Family medicine (n=748)	510 (68)	1	N/A	—	N/A
		Pediatrics (n=299)	192 (64)	0.84 (0.63-1.11)	.22	—	N/A
	**Practice type**						
		Solo or group (n=696)	450 (65)	1	N/A	1	N/A
		Other (n=351)	252 (72)	1.39 (1.05-1.84)	.02	1.16 (0.83-1.61)	.39
	**Part of clinical system**						
		No (n=457)	280 (61)	1	N/A	1	N/A
		Yes (n=590)	422 (72)	1.59 (1.22-2.06)	<.001	1.33 (0.98-1.79)	.07
	**Rurality**						
		Urban (n=363)	258 (71)	1	N/A	1	N/A
		Suburban (n=525)	350 (67)	0.81 (0.61-1.09)	.17	0.87 (0.63-1.19)	.38
		Rural (n=159)	94 (59)	0.59 (0.40-0.87)	.008	0.59 (0.39-0.90)	.02
	**Region**						
		Northeast (n=265)	161 (61)	1	N/A	1	N/A
		Midwest (n=247)	167 (68)	1.35 (0.94-1.94)	.11	1.35 (0.92-1.99)	.13
		South (n=333)	231 (69)	1.46 (1.04-2.05)	.03	1.73 (1.20-2.49)	.003
		West (n=202)	143 (71)	1.57 (1.06-2.31)	.03	1.52 (1.01-2.31)	.047
	**COVID-19–related financial strain**						
		None or a little (n=360)	241 (67)	1	N/A	—	N/A
		Moderate or more (n=687)	461 (67)	1.01 (0.77-1.32)	.96	—	N/A

^a^PCPs: primary care professionals.

^b^OR: odds ratio.

^c^N/A: not applicable.

^d^The variable was not included in the multivariable model because it was not statistically significant at the bivariate level.

## Discussion

### Principal Findings

The findings of our national study suggest that adolescent telehealth has achieved widespread adoption in the year since the COVID-19 pandemic began, with most PCPs in our national study reporting that they used adolescent telehealth and wanted to keep using it. The vast majority (89%) of PCPs reported using telehealth to see adolescents in the prior 3 months, including for chronic disease management, acute care, and mental health. This level of adoption is far higher than that reported in prepandemic studies, which found that very few pediatricians or family physicians were using telehealth to deliver care (13% and 15%, respectively) [9,10]. On average, the PCPs in our sample reported that adolescent telehealth offered more advantages than disadvantages, with advantages including increased access to care and reduced time and transportation burdens for families. The most commonly noted disadvantages were the inability to perform physical examinations and technology problems. Despite these limitations, PCPs indicated broad support for adolescent telehealth after the pandemic is over, with approximately two-thirds wanting to offer such visits themselves. This support suggests a pressing need to build on the strengths, address the challenges, and evaluate the quality of adolescent telehealth to ensure it remains a viable option for primary care delivery in the postpandemic era.

Our study provides novel data on two potential challenges for the delivery of adolescent telehealth: vaccine communication and privacy. With regard to vaccine communication, we found that the proportion of PCPs reporting that they “always” recommended vaccines during adolescent telehealth visits was high for seasonal influenza vaccine, perhaps in response to concerns about an influenza–COVID-19 “twindemic.” Consistent recommendations were less common for HPV, Tdap, and meningococcal vaccines. In the case of the HPV vaccine, prepandemic studies that used similar measures in the context of traditional primary care documented levels of recommendation consistency that are comparable to or lower than what we observed [11-13]. Although such points of comparison are not available for other adolescent vaccines, our findings suggest that many PCPs are including vaccine counseling in adolescent telehealth visits, and these data can inform interventions to support and further strengthen this communication. For example, PCPs may benefit from electronic health record prompts to remind them to recommend adolescent vaccines during telehealth visits and to counsel families about how to schedule those visits. This care coordination could help to ensure that adolescent telehealth complements rather than competes with the in-person care that is integral to the delivery of vaccinations and other routine preventive health services for adolescents [14].

Consistent with prior studies [5,6], our findings suggest that privacy constitutes an important consideration but may not be a primary barrier to the delivery of adolescent telehealth. Among the PCPs in our sample with recent experience, only approximately one-quarter perceived a lack of privacy as a disadvantage of adolescent telehealth. At the same time, however, only approximately one-quarter routinely offered adolescents confidential time during telehealth visits. Despite being a recommended practice, confidential time is also inconsistently offered during in-person visits [15,16]; therefore, this low level of guideline adherence is perhaps unsurprising. Nevertheless, PCPs may have the opportunity to strengthen their telehealth practice by more consistently offering confidential time and by counseling adolescents to take steps that may help protect their privacy, such as using earphones [5,7,17].

Our study can inform future policy making by documenting PCPs’ support for offering adolescent telehealth after the COVID-19 pandemic is over. Telehealth adoption during the pandemic has been possible due to expanded payer reimbursement, which prepandemic studies identified as the single largest barrier to bringing pediatric telehealth programs to scale [9,14,18]. Importantly, most PCPs in our sample believed that health insurance plans should continue to reimburse for adolescent telehealth after the pandemic is over. Furthermore, many PCPs believed that families should have the option of telehealth for adolescent primary care visits and wanted to offer telehealth visits themselves. Wanting to offer adolescent telehealth was more common among PCPs who had recently used it or who worked in the South or West regions of the United States, but it was less common among those serving rural areas. Future research can extend the present study by assessing factors, such as internet connectivity, that might explain these geographic differences.

### Strengths and Limitations

This study is, to our knowledge, the first national evaluation of PCPs’ experience using adolescent telehealth in the context of the COVID-19 pandemic. Study strengths include the use of data from a large, national sample of PCPs, including physicians, advanced practitioners, and nurses. Although evaluating adolescent telehealth from the perspective of those who deliver it is a study strength, our use of self-reported measures also constitutes a limitation. For example, PCPs may have overestimated the consistency with which they recommended vaccines or offered confidential time during adolescent telehealth visits. Another limitation is the modest response rate among advanced practice providers and nurses. Including nonphysician participants was important for enriching our data with diverse perspectives, but additional research with larger sample sizes may be needed to more fully understand how adolescent telehealth practice and attitudes vary across clinical roles. We acknowledge that other perspectives and data sources are important for understanding the impact of adolescent telehealth. Most notably, PCPs in our sample perceived telehealth as expanding access to care and reducing burden on their patients, but future research is needed to understand the extent to which adolescents and their families experience telehealth as patient-centered and equitable. Finally, we note that our study examines telehealth broadly as including visits by video and telephone. PCPs’ experience of adolescent telehealth may vary between these two delivery modes as well as by health system factors, such as the extent to which telehealth is integrated with electronic health record platforms and patient portals. Future research will be needed to understand the influence of these contextual factors.

### Conclusion

One year into the COVID-19 pandemic, our national study finds that PCPs have widely adopted adolescent telehealth and endorse its continued use. In light of this endorsement, health care system leaders, payers, professional organizations, researchers, and other key stakeholders should redouble their efforts to support PCPs in adolescent telehealth delivery, including by further evaluating and fairly reimbursing such services. In this way, we can ensure that telehealth realizes its potential to increase health care access and to serve adolescents in a way that is effective, patient centered, and equitable.

### Data Availability Statement

Deidentified individual participant data will not be made available.

## Abbreviations

HPV: human papillomavirus
PCP: primary care professional
Tdap: tetanus, diphtheria, and acellular pertussis
